# Temporal variation of residential pesticide use and comparison of two survey platforms: a longitudinal study among households with young children in Northern California

**DOI:** 10.1186/1476-069X-12-65

**Published:** 2013-08-20

**Authors:** Xiangmei (May) Wu, Deborah H Bennett, Beate Ritz, Daniel J Tancredi, Irva Hertz-Picciotto

**Affiliations:** 1 Department of Public Health Sciences, University of California, Davis, CA, USA; 2 Department of Public Health Sciences, University of California, Los Angeles, CA, USA; 3 Department of Pediatrics, School of Medicine, University of California, Davis, CA, USA

**Keywords:** Residential pesticide, Use frequency, Telephone survey, Web survey, Temporal variation, Within-household variation

## Abstract

**Background:**

Pesticide use patterns are essential inputs into human pesticide exposure models. Currently, data included for modeling purposes have mostly been collected in cross-sectional surveys. However, it is questionable whether responses to one-time surveys are representative of pesticide use over longer periods, which is needed for assessment of health impact. This study was designed to evaluate population-wide temporal variations and within-household variations in reported residential pesticide use patterns and to compare alternative pesticide data collection methods – web surveys versus telephone interviews.

**Method:**

A total of 481 households in Northern California provided up to 3 annual telephone interviews on residential pesticide use; 182 of these households provided up to 6 quarterly web surveys that covered the same topics for some of the same time periods. Information on frequency and areas of application were collected for outdoor and indoor sprays, indoor foggers, professional applications, and behind-the-neck treatments for pets. Population-wide temporal variation and within-household consistency were examined both within telephone surveys and within web surveys, and quantified using Generalized Estimating Equations and Mixed Effect Modeling. Reporting between the two methods, the telephone survey and the web survey, was also compared.

**Results:**

Use prevalence of outdoor sprays across the population reported in both the annual telephone surveys and the quarterly web surveys decreased over time, as did behind-the-neck treatment of pets reported in the quarterly web survey. Similarly, frequencies of use of these products decreased in the quarterly web surveys. Indoor sprays showed no statistically significant population-wide temporal variation in either survey. Intraclass correlation coefficients indicated consistent use within a household for behind-the-neck treatment on pets and outdoor sprays but great variability for the use of indoor sprays. Indoor sprays were most consistently applied in the bathroom and kitchen. Outdoor sprays were consistently more often applied by male household members, while indoor sprays were not. The two survey approaches obtained fairly similar results on the prevalence of using pesticides, but found discrepancies in use frequencies. In addition, the number of products purchased was positively correlated with application frequency for outdoor sprays (R = 0.51, *p* = 0.0005) but not for indoor sprays.

**Conclusions:**

In this population, repeated surveys are necessary either to obtain a reliable estimate of the average household use of pesticides or to project potential temporal changes of pesticide use. Web surveys could collect comparable data to traditional telephone surveys for some information. However, researchers need to consider the internet acceptability among the target population and balance lower participant burden against the need for sufficiently accurate time-varying measurement, to improve subject retention in longitudinal surveys.

## Background

According to several national and regional pesticide surveys, a majority of U.S. households use pesticides in their house, garden or yard [1-5]. Residential pesticide use has been identified as the most important contributor to children’s exposure to pyrethroid insecticides, currently widely used indoors and in residences in the U.S. [6]. Exposure to pyrethroid pesticides may alter early neurologic and reproductive development in fetuses and infants [7-9], affect adults’ neurologic system, immune system, and reproductive functions [10-13], and might increase the risk of breast cancer [14,15]. To estimate human exposures and risks attributable to residential pesticide applications, detailed information on pesticide use patterns, such as the type of application, frequency, and place/room treated are essential inputs for human exposure models that feed into risk assessment [16].

Recent information on pesticide exposure related behaviors has mostly been collected in cross-sectional surveys [1-3,17,18]. Administration of a one-time questionnaire is economical and widely used for estimating pesticide exposure in epidemiology studies. Nevertheless, it is generally difficult for participants to recall and report past uses and behaviors accurately, especially behaviors they engaged in a long time ago [19,20]. By collecting longitudinal data in a prospective manner, one can assess whether one-time surveys of short-term behavior are adequate for capturing these same behaviors over longer time periods. Longitudinal data are also ideal for improving our understanding of within-household temporal variability of pesticide use. To date, longitudinal data on the frequency and consistency of residential pesticide use are very limited. Berkowitz et al. observed temporal variations for urinary phenoxybenzoic acid (PBA) among pregnant women in New York City consistent with seasonal spraying of pyrethroid pesticides [21]. However, they did not collect data on residential pesticide use, possibly because their study participants mostly lived in apartments, where tenants may not be aware as to whether the apartment owner hired a pest control company to apply pesticides. The NHEXAS study reported on chlorpyrifos concentrations in dust over a one-year measurement period, but did not assess pyrethroid pesticides or collect data on pesticide usage patterns [22]. Due to their prolonged half-life in indoor environments, pesticides in house dust may persist over extended periods [23], thus dust measurements may not reflect recent use patterns. More data is needed to confirm the reliability of using cross-sectional surveys to estimate long-term exposure, and in modeling potential exposures to pesticides based on self-report of use and health effects from cumulative exposures over extended time periods.

As part of the USEPA-funded Study of Use of Products and Exposure-Related Behavior (SUPERB) [24], information on pesticide use patterns in households with young children was collected during three annual telephone interviews and also in up to six quarterly web surveys. We previously reported our cross-sectional data for residential pesticide use collected during the first year telephone interview [18]. Here we examine longitudinal variations in pesticide use patterns to evaluate the reliability and validity of one-time questionnaires used in epidemiologic studies. In addition, we compared data we collected in annual telephone interviews and quarterly web surveys to gain insights into the reliability of this new collection platform and its promise for use in future epidemiological studies.

## Methods

### Data collection

An overall goal of the SUPERB study was to determine factors that influence environmental exposures to young children focusing on pesticides along with other chemicals in consumer products. Children are more vulnerable to pesticide toxicity in early development and behaviors such as increased hand to mouth activity and crawling on floors and carpets result in greater exposures and internal doses [8,25,26]. Candidate households were randomly selected from the birth certificate records of children born between 2000 and 2005 in twenty-two counties in the greater Sacramento and San Francisco Bay Area and surrounding counties. The enrollment rate for the telephone survey was 25.5%, enrolling 499 out of 1,955 families contacted. Further recruitment details can be found in Hertz-Picciotto et al. [24]. A total of 481 households in Northern California completed the section on residential pesticide use in at least one of the annual telephone interviews. Telephone interviews were attempted annually for each household in three consecutive years between October 2006 and March 2010. Note that we did not track if participants moved from one home to another during the study. Interviews were conducted in Spanish or English, by trained interviewers, many of whom were bilingual and bicultural, and data collected in telephone interviews were entered into a web database either during interviews, or from paper forms after the interview. For a portion of the study period, i.e. between October 2007 and September 2009, residential pesticide use data were also collected in quarterly web surveys from a subsample of 182 households randomly recruited from the telephone survey participants, with a higher enrollment rate of 77%. For the web surveys, we only targeted participants able to complete surveys in English, and thus do not know how receptive the non-English-speaking participants would be to completing the survey. Participants completed the web surveys over an 18- or 15-month period (25 participants joined the study too late to complete the 18-month period). Participants (N = 12) who lacked a computer or Internet service but agreed to participate were offered equipment and online services and an in-person orientation about computer use and the web survey. Not every participant completed all longitudinal surveys in either telephone or web data collection.

We collected application frequency for outdoor and indoor sprays, indoor foggers, and professional applications, and behind-the-neck treatments for pets. The frequency information was obtained for the year prior to the telephone interview and for the three months prior to the web survey. Additional details such as size of application area and rooms treated were obtained for the most recent applications only, both in the phone and web surveys. We also elicited demographic characteristics, use of personal care and household cleaning products, dietary intake, and tobacco use/exposures [27-29].

Research protocols and consent forms of this study were approved by the Institutional Review Board of the University of California at Davis. A detailed description of the design and approach of the overall SUPERB study can be found in Hertz-Picciotto et al. [24].

### Data analysis

Descriptive statistics on survey completion rate, pesticide use, and use frequency are reported separately for each survey mode, telephone interviews and web surveys. Regression methods for longitudinal data were used to characterize explainable variation in pesticide usage reports and to estimate within-household correlation coefficients based on between-household and within-household residual variance components. Longitudinal models were fit on data from households that completed more than one survey of the same mode.

For each survey mode and type of pesticide application, a separate logistic regression model assessed explainable variation in the dependent variable use (Yes vs. No) attributable to interview wave (first, second or third of three annual telephone surveys and from first to sixth wave of the web surveys), dwelling type (single family house (SFH) vs. other) and, for the quarterly web surveys, a binary indicator for season (November thru April, the cooler season in Northern California, versus May thru October, the warmer season). For each outcome, the interview wave was evaluated as a continuous covariate to assess monotonic trends. Logistic regression models were estimated using Generalized Estimating Equations with an exchangeable within-household residual correlation structure [30].

For the use frequency outcomes, mixed-effects linear regression models were used to assess interview wave and, for the web survey, season effects. In addition, these models were used to assess the within-household consistency of household usage frequency relative to other households. Because of the highly skewed distributions of usage frequency outcomes, we rank-transformed them prior to fitting the regression models [31]. We then used Kendall’s intraclass correlation coefficients (ICC) of the rank-transformed variables to characterize within-household consistency and computed as the ratio of the between-household residual variance component to the sum of the between-household and within-household residual variance components [32,33].

Further, we compared the pesticide information collected in both telephone and web surveys from the same participants during the approximately same time period. Specifically, we matched the data obtained from the web surveys (conducted between October 2007 and September 2009) to the data from Year-2 telephone interviews (conducted between April 2008 and February 2010) that elicited information from the same household, which ensured the maximum overlap of time periods covered by both surveys. In the telephone surveys, application frequency was reported as use during the warm and cool seasons and these frequencies were summed to obtain frequency of use per year and average use per 3-month period. In the web surveys, we summed the reports of use frequency (for 3 month periods) across all periods and divided by the total length across all reporting periods to convert to use per year and average use per 3-month period. Application frequency was grouped as: 0–1, 2–3, and 4 or more times during each 3-month period of reporting. For each type of application, we compared the two survey modes only for households that ever reported an application in either the telephone interview or web survey. We calculated percent agreement among households in which the telephone survey response was in a specified category (e.g., 0–1 time every 3 months). Consistency of categorical usage reports from the same household were characterized using Cohen’s kappa, a measure of agreement corrected for chance [34].

All analyses were performed using SAS Version 9.2. Statistical significance testing was done using an alpha-level of 0.05 (two-sided) to define a difference as statistically significant, unless otherwise noted.

## Results

### Demographics and survey completion rate

Since the web survey families were recruited from those who enrolled in the telephone surveys, we compared participants in both with participants in only the telephone interviews. The age and sex distributions were similar, but the web survey participants were more educated, and a higher percentage were non-Hispanic Caucasians (Table 1), possibly due to the lack of a Spanish version for the web surveys. Factors that may influence pesticide use, such as home ownership, building type, and neighborhood type, are also shown in Table 1.

**Table 1:** Demographics of survey participants per household in northern California

	**Telephone interview**	**Web survey**
N	481	182
**Sex** - female	401 (83%)	153 (84%)
**Age** (Median: year)	37	36
**Race/Ethnicity**		
White (not Hispanic)	262 (55%)	117 (65%)
Asian (not Hispanic)	57 (12%)	17 (9%)
Black (not Hispanic)	15 (3%)	4 (2%)
Other (not Hispanic)	42 (9%)	21 (11%)
Hispanic (all races)	102 (21%)	23 (13%)
**Education**		
High school or lower	99 (21%)	19 (10%)
College degree or some college	262 (55%)	107 (59%)
Master, Doctor, and professional degree	118 (25%)	56 (31%)
**Job Status**		
Employed	233 (49%)	99 (54%)
Unemployed (including stay-at-home parents)	188 (39%)	67 (37%)
Other	38 (8%)	13 (7%)
Missing	20 (4%)	3 (2%)
**Homeowner**	349 (73%)	140 (77%)
**Building Type**		
Single house detached	395 (82%)	160 (88%)
Single attached house	38 (8%)	12 (7%)
Apartment	46 (10%)	10 (5%)
**Neighborhood Type**		
Commercial	12 (3%)	1 (1%)
Residential	399 (83%)	157 (86%)
Rural	23 (5%)	8 (4%)
Combination of above	43 (9%)	16 (9%)
**Number of Children in the Household** (median)	2	2

The percentage of households that completed telephone interviews and web surveys are summarized in Table 2. Not all participants completed all of the assigned interviews or web surveys. A total of 51% of participants completed all three annual telephone interviews, while only 38% of web survey participants completed 5 or 6 web surveys. In addition, pesticide users had higher compliance to web surveys, with 46% completing 5 or 6 surveys, as compared with 11% of non-users.

**Table 2:** Completion of annual phone interviews and quarterly web surveys

**# of surveys completed**	**Among all households**	Among users*
	**N (%)**	**N (%)**
Phone interview	N = 481	N = 429
3	245 (51%)	223 (52%)
2	90 (19%)	81 (19%)
1	146 (30%)	125 (29%)
Web survey	N = 182	N = 136
5 or 6	68 (38%)	63 (46%)
4	19 (10%)	19 (14%)
3	19 (10%)	12 (9%)
2	24 (13%)	17 (13%)
1	52 (29%)	25 (18%)

*Users of any types of pesticides.

### Prevalence of using pesticides

**Figure 1:**
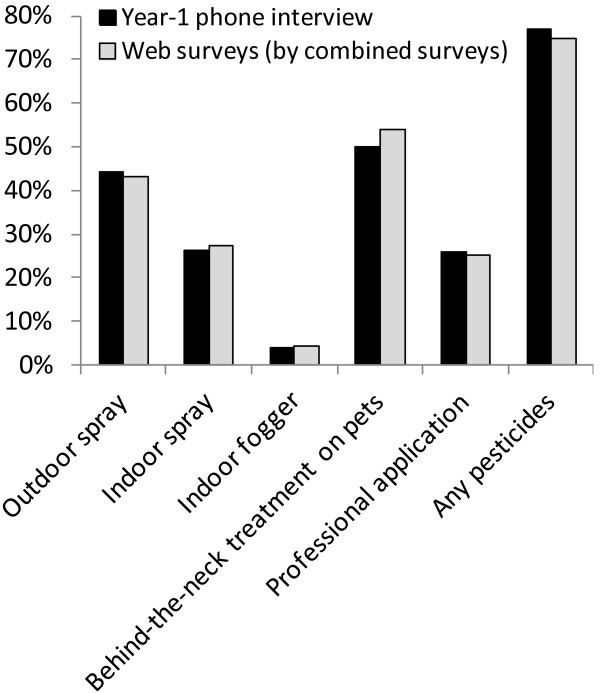
**Prevalence of using pesticides: responses in the first-year telephone interviews (N = 477) and web surveys (N = 182).** Note: Four of the telephone survey participants did not complete the pesticide section of the interview in the first year but only in a subsequent year. Percentage of behind-the-neck treatment on pets was calculated among pet owners.

Overall, 77% and 75% of the participants reported using pesticides in their residences in the Year-1 telephone interview and in the web survey, respectively. The percentage of households reporting applying a given type of pesticide (in the year before the interview) in the Year-1 telephone interview, an example of cross-sectional survey data, and the percentage reporting an application of that same type in at least one of their web surveys (total recall period covered 13.2 months on average) were also similar (Figure 1).

The consistency of pesticide use in the quarterly web surveys is presented in Table 3. Among individual households using pesticides, most appear to use them intermittently, with few reporting pesticide applications in all surveys. The majority of users reported using outdoor and indoor sprays in half or fewer of the surveys completed, while behind-the-neck treatments on pets appeared to occur more consistently.

**Table 3:** Consistency of pesticide use reported by users in completed 3-month web surveys

	**N of users**	**Used in <50% of the surveys**	**Used in 50% of the surveys**	**Used in >50% but not all surveys**	**Used in all surveys (>1 survey)**	**Used in the only survey completed**
Outdoor spray	78	42(54%)	12(15%)	7(9%)	12(15%)	5(6%)
Indoor spray	50	27(54%)	8(16%)	1(2%)	3(6%)	11(22%)
Indoor fogger	8	5(62.5%)	2(25%)	1(12.5%)	—	—
Behind-the-neck treatment on pets	55	7(13%)	7(13%)	14(25%)	15(27%)	12(22%)
Professional application	26	9(35%)	11(42%)	6(23%)	—	—

Population-wide longitudinal variations in using pesticides in both the quarterly web and annual telephone surveys were examined using GEE models. In the web surveys, the prevalence of use of outdoor sprays showed a decreasing temporal trend (p = 0.005), and use in the warm season was higher than in the cool season (p = 0.002). Decreasing likelihood of use was also observed for behind-the-neck treatment on pets (p = 0.01), but no seasonal variation was observed. We note that pet ownership did not decrease over time. For indoor sprays and professional applications, no longitudinal or other variation was observed. The use of indoor fogger was too rare to examine.

For the telephone surveys, we observed a statistically significant decreasing likelihood of using outdoor sprays over years (p = 0.021) across the population. We also found that participants who lived in a SFH were more likely to use outdoor sprays than those who lived in an apartment (p = 0.029). No statistically significant variation was observed for indoor sprays, indoor foggers, and behind-the-neck treatments for pets.

We compared responses to telephone interviews and web surveys for 141 households who participated in both surveys during approximately the same period. The majority (74-97% depending on the type of pesticide application) of the households provided the same answers for whether or not they applied certain pesticides in the past year in each of the two types of surveys (Table 4). However, almost a fifth of all participants who answered that their households were not using pesticides in the telephone survey, 19% for outdoor sprays and 13% for indoor sprays, reported usage in the corresponding web surveys. Conversely, 6% and 13% of households reported having used outdoor or indoor sprays in the telephone survey but not in the corresponding web surveys. Kappa coefficients (κ) were 0.86 for behind-the-neck treatments for pets and professional applications, suggesting good agreement, but were lower for outdoor sprays (κ = 0.50) and indoor sprays (κ = 0.36), indicating fair to moderate agreement in reporting these types of applications across survey methods at the individual household level.

**Table 4:** Consistency of prevalence reported in telephone interviews and web surveys

**Reponses in Year-2 phone interviews**^**a**^	**Reponses in web surveys**		**Kappa coefficient**
	**User**	**Non-user**	
**Outdoor spray (N = 140)**			0.50
User	30%	6%	[0.36,0.64]
Non-user	19%	45%	
**Indoor spray (N = 141)**			0.36
User	15%	13%	[0.19,0.53]
Non-user	13%	60%	
**Indoor fogger (N = 141)**			0.74
User	4%	1%	[0.49,0.98]
Non-user	1%	93%	
**Behind-the-neck treatment on pets (N = 76)**^**b**^			0.86
User	55%	5%	[0.75,0.98]
Non-user	1%	38%	
**Professional application (N = 97)**^**c**^			0.87
User	24%	4%	[0.75,0.98]
Non-user	1%	71%	

^a^Only the data from Year-2 telephone surveys (conducted between April 2008 and February 2010) were used for comparison, as this resulted in the most overlap in time between both surveys (web surveys were conducted between October 2007 and September 2009). ^b^Among pet owners only. ^c^Questions on professional applications were added in the middle of the study.

### Frequency of pesticide applications

**Figure 2:**
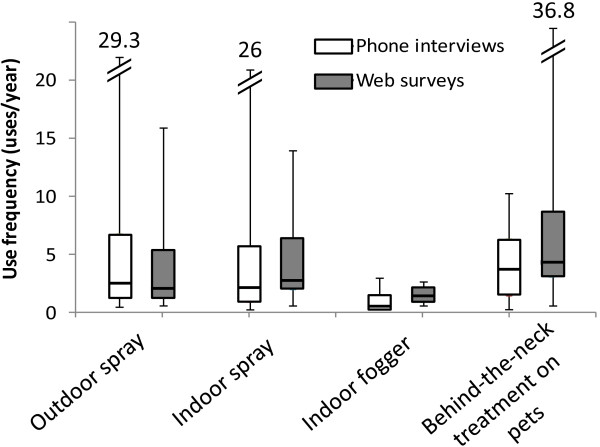
Distribution of frequency of pesticide applications reported in the telephone and web surveys.

We recorded the frequency of each type of pesticide application “in the past year” for the annual telephone interviews and “in the past three months” for the quarterly web surveys. Figure 2 outlines the distribution of the application frequencies among users who completed 2 or 3 years of telephone surveys and those who completed 4 to 6 web surveys. The reported use frequencies collected with the two survey approaches fell into similar ranges except for a few outliers.

Mixed-effects models for the rank-transformed pesticide use frequencies allow us to assess population-wide interview wave effects and season effects as well as within-household consistency (Table 5). In the quarterly web surveys, controlling for season, we observed differences by interview wave for reported application frequencies of outdoor sprays (p < 0.001), and higher frequencies in the warm than in the cool season (p < 0.001). A decreasing trend across quarters was also observed for behind-the-neck treatments on pets (p = 0.002). No statistically significant seasonal or longitudinal variation was observed for indoor sprays or professional applications. Kendall’s ICCs, used for evaluating within-household consistency, were high for professional application (ICC = 0.77) and pet treatment (ICC = 0.66), but relatively low for self-application of outdoor (ICC = 0.30) and indoor sprays (ICC = 0.20). The ICCs suggest consistent use of professional applications and pet treatments and greater variability of indoor spray treatments within households.

**Table 5:** Temporal variation of pesticide use frequency and Kendall’s intraclass correlation coefficients (ICCs)

**Type of pesticide application**	**Telephone survey**		**Web survey**	
	**Effect**	**ICC**	**Effect**	**ICC**
Outdoor sprays	No variation observed	0.16	Decreasing across quarters	0.30
			warm season > cool season	
Indoor sprays	No variation observed	0.05	No variation observed	0.20
Behind-the-neck treatment on pet	No variation observed	0.45	Decreasing across quarters	0.66
Professional application in the yard	—	—	No variation observed	0.77

Note: Results from mixed-effects linear regression models of rank-transformed longitudinal usage frequencies. In annually telephone surveys, pesticide use frequencies were tested for variation across years, and analyses were conducted among users who completed more than one surveys. In quarterly web surveys, pesticide use frequencies were tested for seasonal variation and variation across quarters, and analyses were conducted among users who completed more than one surveys.

In contrast, no statistically significant longitudinal variation across the population was observed for any type of pesticide applications in the annual telephone surveys. Additionally, the ICCs were much lower than those for the quarterly web surveys, indicating greater variability of use patterns within individual households on a yearly basis than on a three-month basis. The ICC for behind-the-neck treatments on pets was higher than for outdoor and indoor sprays, consistent with the web survey findings.

The comparison of individual household second year telephone interviews and web surveys conducted during the same time frame covers all households who participated in both surveys (Table 6). For households having a low frequency of use, 68-90% of households, depending on type of application, reported similar use frequencies in both survey approaches. More inconsistency was found between telephone interviews and web surveys for those with higher use frequencies, for instance, 6 out of 7 households who reported medium application frequency (on average 2–3 time in a 3-month period) for outdoor spray in telephone interviews were low frequency users based on the web surveys. We obtained Kappa coefficients below 0.25, indicating the agreement between use frequencies reported in two types of surveys were rather poor.

**Table 6:** **Consistency of use frequency reported in telephone interviews (converted to average use in a 3-month period) and web surveys (household average use in 3-month recall period)**^**a**^

**Reponses in Year-2 telephone interviews (average use frequency for a 3-month period)**^**b**^	**Reponses in web surveys (household average # of uses in a 3-month period)**			**% of web surveys that agreed with telephone surveys**	**Kappa coefficient**
	**0-1**	**2-3**	**4 or more**		
**Outdoor spray (N = 77)**					0.22
0-1	74%	9%	3%	86%	[-0.01,0.46]
2-3	8%	1%	0%	17%	
4 or more	0%	3%	3%	50%	
**Indoor spray (N = 56)**					0.06
0-1	70%	9%	4%	85%	[-0.15,0.28]
2-3	11%	2%	0%	17%	
4 or more	2%	4%	0%	0%	
**Indoor fogger (N = 10)**					—
0-1	90%	10%	0%	90%	
**Behind-the-neck treatment on pets (N = 45)**					0.17
0-1	51%	20%	4%	68%	[-0.06,0.41]
2-3	9%	7%	7%	30%	
4 or more	0%	0%	2%	100%	

^a^Note that only those who have reported use either in telephone interviews or in web surveys were included. Frequencies of professional applications were not compared, since the location categories do not match for telephone interviews and web surveys.

^b^This was obtained by dividing the annual use by 4.

We also asked the numbers of outdoor and indoor spray products households purchased in each web survey, and added them up to examine correlations with application frequency over the whole web survey period. We found that the number of outdoor spray products purchased was positively correlated with application frequency of outdoor sprays (R = 0.51, p = 0.0005), while the number of indoor spray products purchased was not correlated with application frequency of indoor sprays, or with frequency of pests treated indoors.

### Place and extent of application

Extent of application – Outdoor sprays were applied on both broad areas and in a spot-wise fashion. Among the 40 households that reported using outdoor sprays more than once in the web survey, 9 applied to broad areas during all applications, and 18 never applied outdoor sprays to broad areas (Table 7). Indoor sprays were applied both in a spot-wise fashion and in cracks and crevices, but not on areas larger than 5 square feet. Among the 16 households that reported using indoor sprays more than once in the web survey, 10 households applied indoor sprays to a single spot, probably where pests were found, in at least half of their applications. These results support what we observed in the cross-sectional data from our first-year telephone interview i.e. that spot application was most common [18].

**Table 7:** Consistency of application extent reported across repeated applications

	**Never applied this way**	**Used in <50% of the applications**	**Used in 50% of the applications**	**Used in >50% of the applications**	**Used in all applications**
	N(%)	N(%)	N(%)	N(%)	N(%)
Outdoor spray (N = 40)					
area application	18(45%)	3(7%)	8(20%)	2(5%)	9(23%)
spot application	15(37%)	5(12%)	11(28%)	4(10%)	5(13%)
Indoor spray (N = 16)					
Extent of application					
One Specific Area (<1 Sqft)	5(31%)	1(6.25%)	4(25%)	3(19%)	3(19%)
Several Specifics Areas (1–5 Sqft)	8(50%)	4(25%)	4(25%)	—	—
Cracks, Crevices, or Edges	11(69%)	1(6%)	2(13%)	1(6%)	1(6%)
Large Area of Room (>5 Sqft)	16(100%)	—	—	—	—
Area applied^a^					
Bathroom	3(19%)	1(6%)	2(13%)	3(19%)	7(44%)
Kitchen	4(25%)	3(19%)	4(25%)	—	5(31%)

^a^Other areas than bathroom and kitchen had small percentage of share and thus were not shown here.

Place of application - Outdoor sprays were most consistently applied to decks, house perimeters, house structures and lawns. Of households that reported applying outdoor sprays more than once in the web survey (N = 41), 34% reported they always sprayed decks, and 27% always sprayed the house perimeter. Indoor spray was most consistently applied in the bathroom and kitchen, with 44% and 31% of households (N = 16) that used these products more than once reporting that they always applied in these places in the web survey.

### Applicators

We asked in the web surveys whether participants applied pesticides themselves or whether their spouse applied them. Among those who reported in at least two web surveys using the same type of pesticides, 18 out of the 41 participants reported that they were the person who applied outdoor sprays; 6 out of the 16 participants applied indoor sprays themselves. Outdoor sprays were consistently more often applied by male household members (63%), while indoor sprays were not, with 25% of households reporting indoor sprays were always applied by male household members, 25% reporting always female household members, and the remainder reporting applications by both males and females. This may contribute to the variability of indoor spray use within households as applications were conducted by two separate individuals. Furthermore, ~80% of participants provided consistent responses about the sex of the applicator in internet surveys and in telephone interviews.

## Discussion

In this study, we collected data on residential pesticide use patterns using two survey approaches: annual telephone surveys and quarterly web surveys. Across the population, we observed decreasing trends of reported uses for outdoor sprays and pet treatments over time as well as great within-household variability for uses of outdoor and indoor sprays. Another finding of this study was that the telephone survey and web survey could collect fairly consistent data on prevalence of pesticide use but not on use frequency. Therefore, web surveys may be able to replace traditional telephone surveys in some cases and result in savings in terms of labor and cost.

### Longitudinal variation of pesticide use patterns

Our data suggest population-wide decreasing temporal trends for the reported prevalence of outdoor spray use in both annual telephone interviews and quarterly web surveys, and for frequency of outdoor spraying and prevalence and frequency of behind-the-neck treatment of pets in both survey modes. Possible explanations include potentially temporary and non-recurrent pest infestations that only required use in earlier years, as we observed higher pesticide use frequencies in individual households at the beginning than the end of the study, or growing participant concerns over adverse health effects from pesticides brought on by our repeated questions about pesticide use, especially among those answering both web and telephone surveys. Lastly, there is a possibility that participants may have neglected to report that they applied pesticides in order to avoid needing to answer more detailed follow-up questions.

As suggested by the low ICC values, the use of indoor spray demonstrates a relatively large amount of within-household variation. Though little temporal variation across the population was observed for indoor spray use, repeated surveys are necessary to obtain a reliable estimate of individual household’s average use. For those with a population-wide decreasing temporal trend, it may be necessary to acquire use information repeatedly over a longer period. Considering that it is hard to keep participants in a study over extended periods, researchers need to balance the requirements for obtaining lengthy follow-up and recall issues. Our telephone interview results show that participants are able to provide relative reliable pesticide use information for the past year. One recall of the past year usage may be less burdensome for participants than four quarterly recalls that covers the same length of time. Another suggestion is that, as the greatest temporal variability resulted from households that had periods of moderate to high use frequencies potentially related to a pest infestation, future surveys might want to include a question on whether or how often there were pest infestations.

In addition, we found that use of outdoor sprays was correlated with the number of products purchased over the whole web survey period, while use of indoor sprays was not. This might be because outdoor sprays were usually applied to broad areas and thus used in larger quantities. In contrast, indoor sprays were usually used for spot and crack and crevice applications i.e. in varied quantity. This should be noted, as sales data have been used as a surrogate for pesticide use and exposure.

### Comparison of survey methods

Web based surveys are a relatively new method of data collection. This method reduces labor costs (particularly in studies of large sample sizes) compared to traditional telephone interviews [35-37], and also reduces human error that may be caused by interviewers mistakes or during data entry [38,39]. Respondents also have more flexibility to complete the survey at a time convenient for them [36]. Therefore, this method is desirable for use in large scale epidemiologic studies, as long as the quality of data can be maintained. However, studies have shown that web surveys work better in educated populations and use may be reduced in populations with limited internet access or computer skills [40-42]. This needs to be considered in designing web survey studies. There has been no study collect longitudinal pesticide use data using web survey. By comparing web survey and telephone survey results obtained in this study, we can gain insights into the reliability of this new method and its promise for use in future epidemiological studies.

The two survey approaches obtained fairly consistent results concerning simple responses such as whether or not pesticides were used. The percentage of households in which pesticides were applied was similar based on quarterly web surveys and our first year cross-sectional interview. Answers from participants who completed both telephone and web surveys showed strong to excellent agreement between the two types of surveys on pesticide use/non-use (74-95% agreement), frequency of use (68-90% agreement for low frequency users), and who applied the pesticide in the home (70-80% agreement).

However, more variation was observed for reported application frequency. Disagreements were observed among moderate and high frequency users, probably due to high variability in the pest problems themselves. The low Kappa coefficients indicated that the agreement for the low frequency use was mostly due to chance. With regard to the comparison across survey platforms, the data were not collected during the exactly same time periods, which may also contribute to the differences we observed.

In terms of subject retention and survey completion, the three annual telephone surveys had better survey completion rates than the six quarterly web surveys (51% vs. 37%). Note that, apart from pesticide use patterns, SUPERB also collected use patterns of other consumer products, food intake frequency, and time activities, and one telephone or web survey could easily take one hour or more, which drastically increased participant burden and may have increased the participant drop-out rate in both types of surveys. As reported in Wu et al., a large number (84%) of web survey participants claimed that they were not able to complete all required quarterly web surveys due to limited time and family responsibility [43].

In summary, web surveys could collect comparable data to traditional telephone surveys in some cases. They may perform better with higher retention rate if participants’ burden could be reduced. As subjects appear to be able to provide reliable data about pesticide use in the past year, considering the low cost and flexibility, one or multiple web surveys to recall the pesticide use in the past year seem to be a sensible choice. However, researchers need to balance participant burden against the need for detail to be collected, in order to improve subject retention in longitudinal web surveys. More broadly, higher drop-outs and missing data in any self-administered data collection platform also must be weighed against the advantages in cost and participant flexibility. Additionally, though not shown in our study, web surveys are usually subject to lower response rate than telephone surveys. One needs to consider the internet accessibility of the target population when deciding to use a web survey. Supplementary means, such as postcard and email reminders, may help improve response rate [40].

### Limitations

First, as discussed in our previous paper evaluating longitudinal time-activity surveys [43], long-term studies with repeated measurement designs are usually subject to large participant loss over time, which reduces the power to evaluate longitudinal variations. To retain a maximum amount of information, we included households that completed two or more rather than all three telephone/web surveys for longitudinal analysis. Second, our respondents are predominantly women, and web survey participants had slightly higher education levels and employment rates compared to telephone survey participants. Based on our data, education level and employment status of respondents did not influence survey completion rate. However, we are uncertain whether there are differences in the reporting of pesticide use between males and females or if higher socio-economic status influences pesticide use. Third, as mentioned above, both our telephone and web surveys included questions about the use of other consumer products, and thus took approximately one hour to complete, which may have been a key reason for the high drop-out rate. Another limitation is that the temporal variations we observed were also influenced by the accuracy of a participant’s recall and compliance, which is hard to quantify and differentiate from real temporal variation. In addition, some of the pesticide application patterns observed in this study, such as the degree of seasonal variation of use frequency, may differ in other geographic areas with a different climate.

## Conclusions

In summary, this study found great within-household variability for indoor spray applications and a population-wide decreasing trend of outdoor spray applications and behind-the-neck treatment on pets. Repeated surveys are needed to characterize residential pesticide use, either to obtain a reliable estimate of the average household use for applications with great within household variation, i.e., outdoor and indoor sprays, or to project longitudinal variation for applications with decreasing temporal trends. Other findings include that, indoor spray was most consistently applied in the bathroom and kitchen; outdoor sprays were consistently more often applied by male household members, while indoor sprays were not; quantity of indoor spray pesticide products purchased may not be a substitute measure of use frequency of indoor spray. Compared with annual telephone surveys, quarterly web surveys provide comparable results concerning the prevalence of pesticide use but showed more variation in application frequencies. Importantly, our web surveys were subject to higher subject attrition. Improvements to minimize participant burden may increase participant retention in longitudinal web surveys.

## Abbreviations

SUPERB: Study of use of products and exposure related behavior.

## Competing interests

The authors declare that they have no competing interests.

## Authors’ contributions

XW was responsible for analysis and interpretation of data and drafting the manuscript, working closely with DB. IHP, DB, and BR made substantial contributions to study design of the SUPERB and data collection, DT contributed to data analysis, and all of them contributed to critical review of the manuscript for intellectual content. All authors read and approved the final manuscript.
